# Supplementary figures and images for: Dynamics of the Gut Microbiome and Transcriptome in Korea Native Ricefish (Oryzias latipes) during Chronic Antibiotic Exposure
Source: Genes (Basel). 2022 Jul 14;13(7):1243. doi: 10.3390/genes13071243 (PMC9322331; doi:10.3390/genes13071243)

Figure S1

(A)

GO biological processes

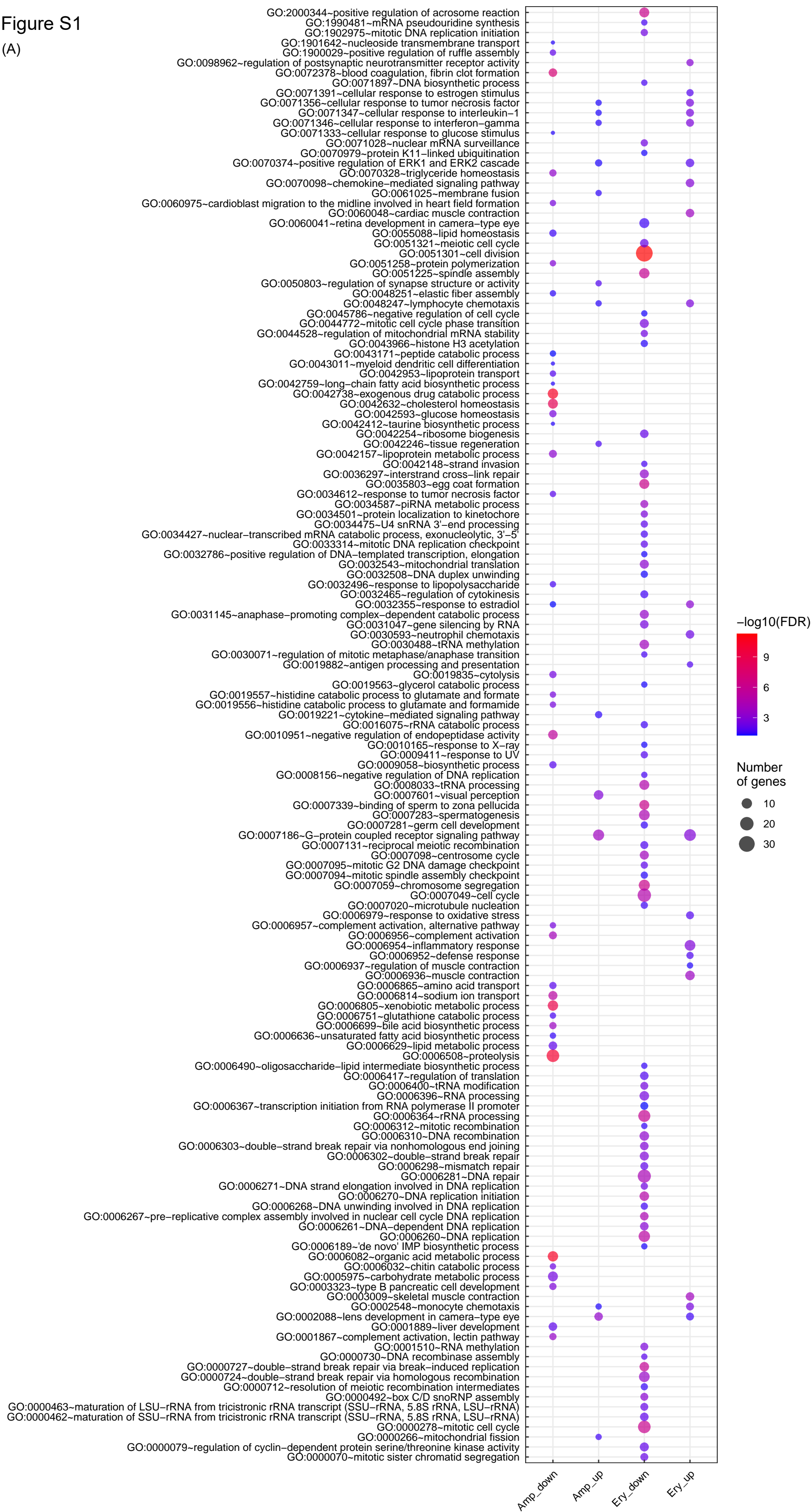

Figure S1

(B)

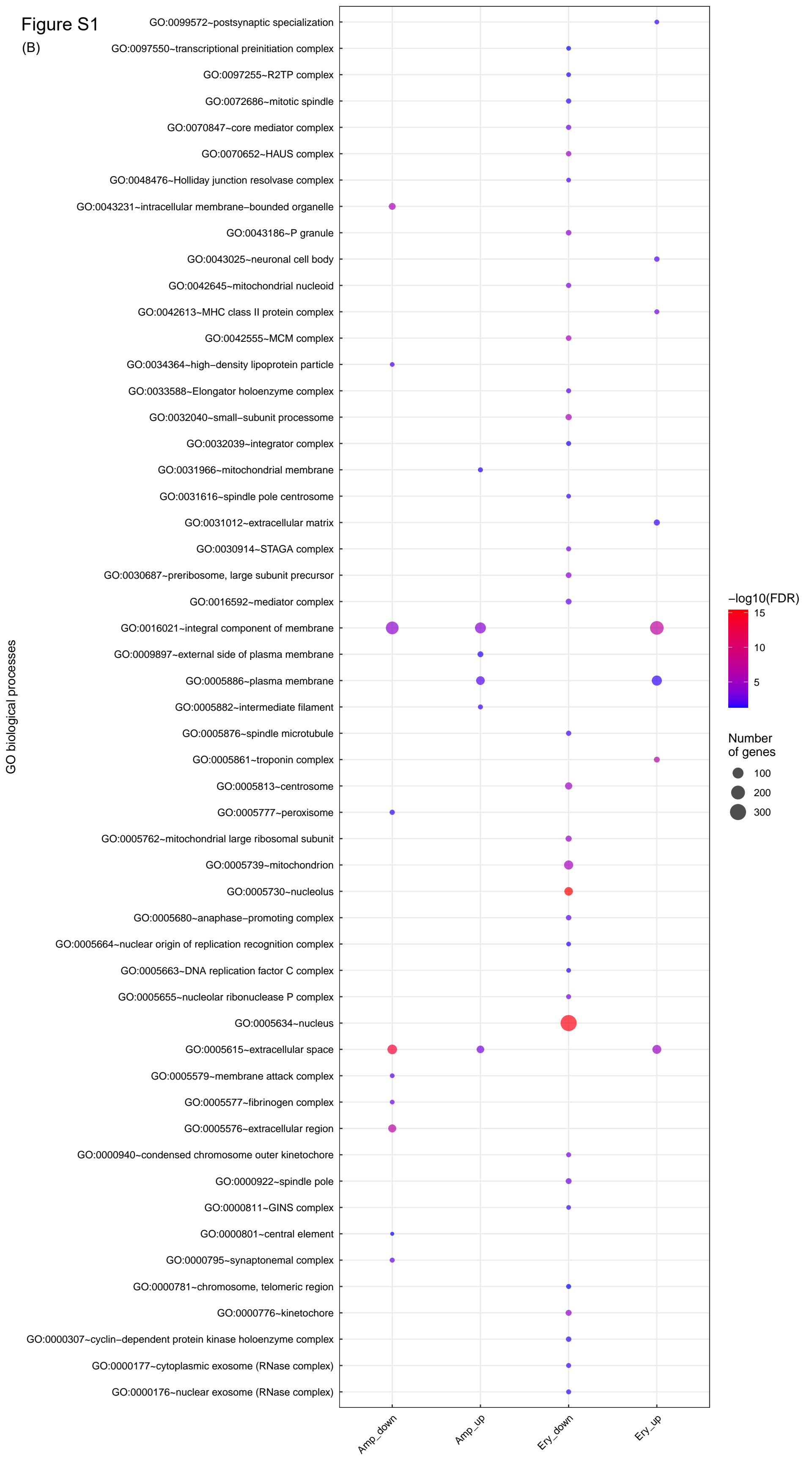

Figure S1  
(C)

GO biological processes

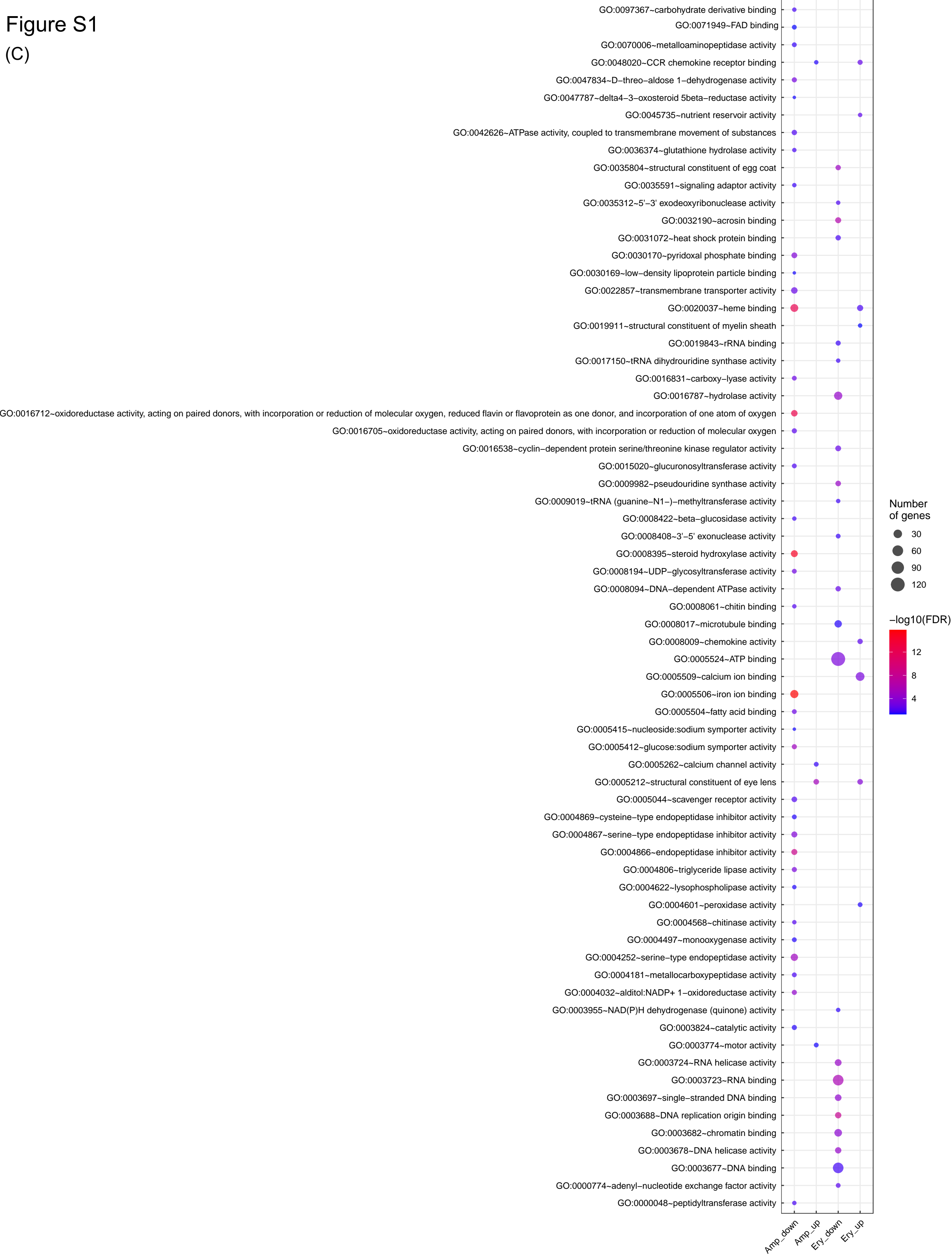

Supplement: Supplementary file 1 [file genes-13-01243-s001.zip › Supplementary Figure S1.pdf]
